# Shortening Psychological Scales: Semantic Similarity Matters

**DOI:** 10.1177/00131644251319047

**Published:** 2025-02-24

**Authors:** Sevilay Kilmen, Okan Bulut

**Affiliations:** 1 University of Alberta, Edmonton, Canada; 2 Bolu Abant Izzet Baysal University, Bolu, Turkiye

**Keywords:** scale abbreviation, semantics, sentence embedding, psychometrics

## Abstract

In this study, we proposed a novel scale abbreviation method based on sentence embeddings and compared it to two established automatic scale abbreviation techniques. Scale abbreviation methods typically rely on administering the full scale to a large representative sample, which is often impractical in certain settings. Our approach leverages the semantic similarity among the items to select abbreviated versions of scales without requiring response data, offering a practical alternative for scale development. We found that the sentence embedding method performs comparably to the data-driven scale abbreviation approaches in terms of model fit, measurement accuracy, and ability estimates. In addition, our results reveal a moderate negative correlation between item discrimination parameters and semantic similarity indices, suggesting that semantically unique items may result in a higher discrimination power. This supports the notion that semantic features can be predictive of psychometric properties. However, this relationship was not observed for reverse-scored items, which may require further investigation. Overall, our findings suggest that the sentence embedding approach offers a promising solution for scale abbreviation, particularly in situations where large sample sizes are unavailable, and may eventually serve as an alternative to traditional data-driven methods.

In psychological testing, many scales exist in both lengthy and abbreviated formats, such as personality assessments. The longer versions, while often more comprehensive, are time-consuming and can be burdensome for both test-takers and administrators (Zheng et al., 2020). This challenge is particularly pronounced when multiple scales within a single instrument are administered together. In such situations, some participants may experience fatigue or disengagement, leading to incomplete responses or insufficient effort in answering all items. This can compromise the quality of the data and the validity of the results.

To address these challenges, various scale shortening (or scale abbreviation) methods have been developed with the aim of reducing the time and effort required for scale administration while maintaining the reliability and validity of the instrument. These methods typically involve selecting a subset of items that capture the key dimensions of the construct being measured without sacrificing too much precision or coverage. Various approaches, such as factor analysis, item response theory (IRT), and machine learning techniques, have been widely used in this context (e.g., Browne et al., 2018; Eisenbarth et al., 2015; Kilmen & Bulut, 2023; Leite et al., 2008; Olaru & Danner, 2021). By streamlining psychological scales, researchers and practitioners can enhance both the efficiency and the accessibility of psychological scales, improving the overall experience for participants and the quality of the data collected (Yarkoni, 2010).

To date, numerous researchers have employed various data analytic techniques to abbreviate lengthy psychological scales, including IRT (e.g., Cooper & Petrides, 2010), genetic algorithms (Kilmen & Bulut, 2023; Sandy et al., 2014; Yarkoni, 2010), and ant colony optimization (e.g., Leite et al., 2008; Olaru et al., 2019; Raborn et al., 2020). While these approaches have proven effective, they often involve complex computational procedures that require large sample sizes and extensive analysis, which can be both demanding and time-consuming for researchers and practitioners. Furthermore, the existing methods for scale abbreviation focus solely on psychometric criteria in selecting the items (e.g., item-total correlation, reliability, and model-data fit); however, they disregard the content aspect of the items. This may lead to shortened scales that are psychometrically sound but highly imbalanced in terms of content.

In contrast, a more practical and efficient alternative for scale abbreviation may lie in the use of sentence embedding techniques, which leverage the semantic similarities between items to guide item selection. In this study, we introduce a novel approach based on sentence embeddings to simplify the scale abbreviation process. Unlike traditional methods, which rely on large datasets to compute item parameters, scale statistics, or fit indices, the proposed method allows for the selection of items based purely on their semantic relationships. By focusing on the meaning of the items rather than their psychometric properties, this approach bypasses the need for administering the scale to a large sample of participants to obtain parameters and statistics for item reduction.

To demonstrate the effectiveness of this approach, we applied it to real-world data and compared it against existing methods used in scale abbreviation, specifically genetic algorithms and ant colony optimization. We selected items for the short form of the Experiences in Close Relationships (ECR) scale (Brennan et al., 1998) using these two automatic item selection techniques alongside our proposed semantic similarity-based method. The results of the abbreviated scales were then compared with the original scale to evaluate the accuracy and efficiency of each method. This study highlights the potential of using sentence embedding as a simple yet powerful tool for scale abbreviation, offering a more accessible and less resource-intensive alternative for researchers and practitioners in psychological measurement.

## Literature Review

### Scale Abbreviation Approaches

In psychological assessments that utilize lengthy scales, some participants may be unwilling to respond to all items, and those with low motivation may even refuse to participate altogether (Zheng et al., 2020). To address these issues, researchers have developed various methods for scale abbreviation to reduce the burden on participants and minimize data loss. The selection of appropriate items for these abbreviated versions is critical, as the short forms must adequately represent the original scales across several key indicators. These indicators differ depending on the abbreviation method employed.

Over the last decade, there has been a growing focus on automatic item selection in scale abbreviation studies. These approaches use different criteria to ensure the shortened scale accurately reflects the content and structure of the original. For instance, genetic algorithms are commonly used in scale abbreviation because they prioritize items that best capture the traits of interest while balancing the cost of additional items. The goal is to maximize the explained variance with fewer items, resulting in a more efficient yet reliable scale (Eisenbarth et al., 2015). By focusing on retaining the most informative items, genetic algorithms help preserve the validity of the original scale while significantly reducing its length (Rachmani et al., 2019; Yarkoni, 2010). Studies that use genetic algorithms to perform different personality inventory abbreviation processes suggest that personality inventories can be significantly shortened without losing validity, and multiple measures can be replaced with a single, brief one. These studies also demonstrate the effectiveness of genetic algorithms in developing short forms from a psychometric perspective (Olaru et al., 2015; Yarkoni, 2010).

Ant colony optimization, another scale abbreviation method, aims to identify items that provide the highest model fit (Leite et al., 2008). This method operates through an iterative process of item selection, where different sets of items are randomly chosen in each iteration. Based on optimization criteria, decisions are made regarding which items advance to the next iteration (Schroeders et al., 2016). These iterations continue until a satisfactory model fit is achieved, often evaluated using criteria such as comparative fit index (CFI), Tucker–Lewis Index (TLI), and root mean squared error of approximation (RMSEA) (e.g., Leite et al., 2008; Olaru et al., 2015; Raborn & Leite, 2018; Schroeders et al., 2016). Ant colony optimization has been successfully applied to create abbreviated versions of widely used assessments, including the Peabody Picture Vocabulary Test (Dunn & Dunn, 1981; Schroeders et al., 2016) and the IPIP-300 Personality Test (Goldberg, 1999; Jankowsky et al., 2020). Although research has demonstrated that both genetic algorithms and ant colony optimization produce efficient, reliable, and valid short versions of scales (Kerber et al., 2022; Olaru et al., 2015; Schroeders et al., 2016), these methods are data-dependent and thus require real data obtained from a representative group of respondents.

Recent studies, however, suggest that natural language processing (NLP) techniques can also be useful in psychometric applications, offering a different route for item selection. For instance, Hsu and colleagues (2018) introduced a novel word embedding-based approach to estimate item difficulty. By calculating the cosine similarity between the vectors of item elements, this method uses semantic features to predict item difficulty. Their findings indicated that this approach could potentially replace pretesting in the future. Building on the work of Hsu and colleagues, we propose that NLP can also be leveraged for selecting items in the scale abbreviation process. In this study, we operationalize sentence embedding techniques to assess the semantic similarity between items. Items with high cosine similarity (a measure of semantic similarity) are considered highly similar, and we introduce the concept of an “item similarity index,” which is the mean cosine similarity of an item compared to all others in the scale. This index serves as a tool for identifying items with similar meanings, which could be used to streamline scales in a more efficient and conceptually grounded manner.

The operationalization of item similarity helps assess how closely an item resembles others in a given scale. Traditionally, items selected for short forms of scales have been chosen through data-driven approaches, with little attention paid to their semantic similarity. This oversight can result in short forms that include items with redundant or missing content, which undermines the goal of capturing a construct in a well-rounded and distinct manner. In psychological assessments, it is crucial to select items that represent different facets of the underlying psychological construct rather than duplicating similar content. In this study, we argue that participants provide similar responses to items with high semantic similarity, which raises concerns about redundancy in scale abbreviation. By focusing on the semantic relationships between items, researchers can create short forms that preserve the diversity of the construct being measured while avoiding unnecessary overlap. Our approach enables the development of short forms that maintain content validity without the need for pretesting, offering a more efficient and conceptually sound method for scale abbreviation.

### Sentence Embedding and Semantic Similarity of Items

Sentence embedding is a well-known NLP technique that converts sentences into vectors of real numbers. This transformation allows sentences that are semantically similar to be represented by similar quantitative vectors (Mishev et al., 2019), enabling the quantitative analysis of textual data. Sentence embedding approaches have been applied in various areas, such as text similarity and text classification (Galal et al., 2024; Shajalal & Aono, 2019). Therefore, sentence embedding is not a new methodology in the field of psychometrics. For example, vectorization techniques have been used in automated scoring studies to evaluate written responses (Ramesh & Sanampudi, 2022).

Various approaches have been recommended for vectorizing textual data. Traditional vectorization techniques include bag of words (BoW), term frequency-inverse document frequency (TF-IDF), and Doc2vec methods (Albitar et al., 2014; Le & Mikolov, 2014; Zhang et al., 2010). While these methods effectively transform text into numerical vectors based on word frequency in a document or corpus, they do not account for the semantic similarity between words. To overcome this limitation, recent studies have proposed Bidirectional Encoder Representations from Transformers (BERT), a transformer-based pre-trained language representation model (Chang et al., 2021; Devlin et al., 2018). BERT learns the contextual relationships within and between sentences through masked language modeling and next sentence prediction during its pre-training process (Arase & Tsujii, 2021).

Building upon the vectorization techniques discussed earlier, converting sentences into numerical vectors enables the calculation of semantic similarities between them. One of the most common methods for measuring the similarity between textual vectors is cosine similarity. The cosine similarity between two vectors A and B is calculated as:

(1) $$\mathrm{Cosine}\;\mathrm{Similarity}=\frac{A\cdot B}{∥A∥\times ∥B∥},$$

where A·B is the dot product of the vectors and ∥A∥ and ∥B∥ are the magnitudes (Euclidean norms) of the vectors. Similar to Pearson correlation, the resulting value for the cosine similarity ranges from −1 to 1 where 1 indicates that the vectors are identical in direction (maximum similarity), 0 indicates that the vectors are orthogonal (no similarity), and −1 indicates that the vectors are diametrically opposed.

The cosine similarity index is widely used in NLP applications such as determining text relevance between documents and detecting plagiarism (Gunawan et al., 2018; Saptono et al., 2018). Moreover, previous research has shown that cosine similarity can be utilized for feature subset selection (Suebsing & Hiransakolwong, 2012). In the context of our study, we consider items as features of a scale. After converting items into vectors using sentence embedding techniques, one can compute the cosine similarity to quantify how semantically similar any two items are. By calculating the cosine similarities between items, we can derive the mean cosine similarity for each item, defined as the average semantic similarity of that item with all other items. Items with lower mean cosine similarity are considered more semantically unique. Selecting items based on low mean cosine similarity ensures that the short form of a scale comprises items that are diverse in content, thereby capturing different facets of the underlying construct. This method allows for the creation of an abbreviated scale that maintains content validity without the need for extensive pretesting.

Calculating semantic similarity indices of items offers several benefits to researchers and practitioners. First, traditionally determining the semantic similarity between item pairs (i.e., through expert evaluation) requires careful analysis by numerous experts over an extended period, making it time-consuming and costly. The scale abbreviation approach based on sentence embedding can automatically assess the semantic similarity of items, thereby reducing the need for expert input and saving both time and resources. Second, by considering the semantic similarity among the items, we can ensure that the short form of the scale includes items that are diverse in meaning. This can prevent overlapping items and maintain the scale’s local independence and content validity. Third, since the proposed scale abbreviation method does not require pre-administration of the scale, it saves additional time and effort for practitioners and researchers.

### Current Study

Despite advances in psychometric techniques, studies on scale abbreviation have not yet incorporated sentence embedding methods. Shortening scales based on psychometric properties, without considering semantic similarities through sentence embedding, may result in the inclusion of items with redundant content in the abbreviated scale. Previous research has demonstrated significant relationships between measures of item similarity and actual inter-item correlations obtained from survey responses (e.g., Larsen & Bong, 2016). This indicates that participants tend to respond similarly to semantically similar items. Thus, including semantically similar items in the short form of a scale may restrict variance along the trait continuum, potentially diminishing the scale’s ability to discriminate between different levels of the construct (Clark & Watson, 1995). Also, redundant items can contribute to participant fatigue or boredom during testing, leading to random responses or nonresponses (Stanton et al., 2002). By integrating sentence embedding techniques into scale abbreviation, researchers can identify and exclude semantically similar items, thereby enhancing the efficiency and validity of the abbreviated scales. This approach not only preserves the content diversity necessary for accurately measuring psychological constructs but also improves the testing experience for participants by reducing redundancy and fatigue.

To overcome the limitations of existing scale abbreviation methods that often ignore semantic similarities between the items, this study proposes a novel approach that utilizes sentence embedding for item selection. By incorporating semantic similarity measures, we aim to create abbreviated scales that maintain content validity while reducing redundancy. We evaluate the effectiveness of the sentence embedding approach by comparing it to two established automatic scale abbreviation methods, which are among the most practical techniques currently available.

Our study investigates the following research questions:

1. Are there significant relationships between IRT item parameters (discrimination and thresholds parameters) and item semantic similarity indices?
2. Does the sentence embedding approach outperform automatic scale abbreviation methods (i.e., genetic algorithm and ant colony optimization methods) in terms of yielding higher information and better model fit indices?
3. What are the correlations between ability (i.e., latent trait) estimates obtained from the original scale and those from the short forms created using the sentence embedding and automatic scale abbreviation approaches?

In the current study, we compare the performance of automatic scale abbreviation methods with the sentence embedding approach. We use BERT as the sentence embedding method to explore the semantic similarities between items. The key reason for focusing on BERT is BERT’s unique ability to generate deep bidirectional representations, which distinguishes it from other unidirectional language models (Devlin et al., 2018). This capacity allows BERT to capture more nuanced semantic similarities between sentences, which is central to the purpose of this study—investigating how semantic similarity impacts the process of scale abbreviation. Studies have demonstrated that BERT excels at sentence-level understanding, making it particularly well-suited for tasks involving semantic analysis (Goldberg, 2019; Manning et al., 2020; Thrush et al., 2020). Another reason for limiting the study to BERT is the aim to conduct an in-depth analysis of its performance and capabilities. Comparisons involving multiple methods can provide broader insights, but they may miss important nuances of individual model behaviors. By narrowing the focus to BERT, we aim to conduct a thorough investigation of its strengths, limitations, and specific contributions to the research questions.

This study compares the novel sentence embedding-based scale abbreviation method with two established techniques: genetic algorithms and ant colony optimization. Genetic algorithms simulate biological processes such as mutation, crossover, and selection to generate optimal solutions for optimization and search problems (Mitchell, 1998). The technique seeks to balance the cost of adding items with maximizing the explained variance of the scale (Kilmen & Bulut, 2023). Similarly, ant colony optimization streamlines the labor-intensive work of scale development and efficiently constructs high-quality short scales from large item pools. This method focuses on maximizing reliability and validity while ensuring a good model fit for the measurement model (Dörendahl & Greiff, 2020; Kerber et al., 2022). Given their prominence in combinatorial optimization, these two methods were chosen as benchmarks to evaluate the performance of the sentence embedding-based scale abbreviation method (Olaru et al., 2015; Schroeders et al., 2016). By addressing these research questions, we aim to demonstrate that incorporating semantic similarity through sentence embedding enhances the scale abbreviation process, resulting in more efficient and psychometrically sound short forms without the need for extensive pretesting.

## Method

### Data Source

The sample of this study consists of 10,798 participants who completed the ECR scale (Brennan et al., 1998). The participants were adults from the United States aged between 18 and 30 (65% female, 35% male). The original data set for the ERC scale is available on the Open-Source Psychometric Project website.^1^

### Instrument

The ECR scale (Brennan et al., 1998) is a 36-item, five-point Likert-type instrument designed to assess attachment-related anxiety and avoidance in romantic relationships. The scale comprises two first-order factors: anxiety and avoidance. The anxiety subscale consists of 18 items (e.g., “I get frustrated if romantic partners are unavailable when I need them”). This subscale measures the extent to which individuals fear rejection and abandonment. The avoidance subscale consists of 18 items (e.g., “I find it difficult to allow myself to depend on romantic partners”). This subscale assesses the degree to which individuals are uncomfortable with closeness and emotional intimacy (see the Appendix A for a complete list of the ECR items). For scoring purposes, one item in the anxiety subscale and nine items in the avoidance subscale are reverse-scored. In our application of the sentence embedding approach, reverse-worded statements were rephrased into their straightforward forms to accurately calculate semantic similarity indices and examine their relationships with item characteristics. For example, the item “I am very comfortable being close to romantic partners” was transformed into “I am not very comfortable being close to romantic partners” to align the semantic direction with other items. Before performing any scale abbreviation procedures, confirmatory factor analysis (CFA) was conducted to validate the two-factor structure of the ECR scale. The results supported the original factor structure, indicating a good model fit with a CFI of 0.948, a TLI of 0.945, and an RMSEA of 0.015. These findings confirm that the ECR scale reliably measures the two distinct dimensions of attachment-related anxiety and avoidance.

### Creating Abbreviated Forms

First, we abbreviated the ECR scale by employing the ant colony optimization approach available in the ShortForm package (Raborn & Leite, 2018) implemented in *R* (R Core Team, 2023). This method selects items for the short form through an iterative process that mimics the foraging behavior of ants to find optimal solutions. In our study, we used the three model fit indices (CFI, TLI, and RMSEA) as stopping criteria for the iterations, with thresholds set at CFI > 0.95, TLI > 0.95, and RMSEA < 0.05. The models were estimated using the Diagonally Weighted Least Squares (DWLS) method. The DWLS method is appropriate for ordinal data, such as Likert-type scales, and provides robust estimates even when the data deviate from normality (Li, 2016). At the conclusion of the abbreviation analyses, we selected nine items per subscale for inclusion in the short form.

Next, we utilized genetic algorithms to create a 9-item short form of the ECR scale. We employed the GAabbreviate package in *R* (R Core Team, 2023), which automates the scale abbreviation process using genetic algorithms. This package builds upon the GA package, leveraging evolutionary algorithms to optimize item selection based on predefined psychometric criteria. The genetic algorithm approach begins by generating an initial population of potential item subsets randomly. Each subset is evaluated using a fitness function that incorporates desired psychometric properties, such as internal consistency (e.g., Cronbach’s alpha), item-total correlations, and model fit indices. The algorithm iteratively evolves the population through processes analogous to natural selection, including selection, crossover, and mutation operations. This evolution aims to maximize the fitness function, thereby identifying the most optimal combination of items for the abbreviated scale. In our implementation, we specified the fitness function to maximize the internal consistency of the subscales while minimizing the number of items, striving for a balance between brevity and reliability. We set the algorithm parameters to target nine items per subscale, aligning with the number of items selected using the ant colony optimization method.

**Figure 1:**
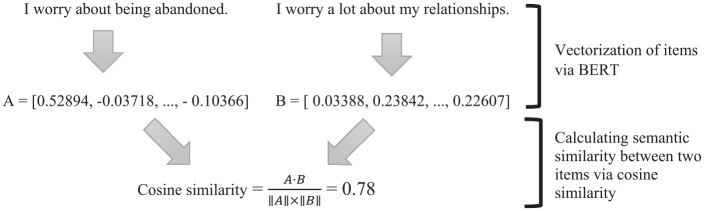
Semantic Similarity Calculation Process via BERT

In the final stage of our data analysis, we applied the sentence embedding approach to perform scale abbreviation. Using Python in the Google Colab environment, we generated vector representations of the ECR scale items. Specifically, we utilized BERT, a pre-trained transformer-based language model, to convert each item into a high-dimensional embedding. BERT produces embeddings consisting of 768 dimensions, so each item was represented by a vector of 768 numerical values (see Figure 1). These embeddings capture the semantic and contextual nuances of the items, allowing us to quantitatively assess the semantic similarities between them. Using the embeddings, we calculated the cosine similarities between each pair of items (Salton & Buckley, 1988) and identified semantically unique items for inclusion in the abbreviated scale.

**Figure 2:**
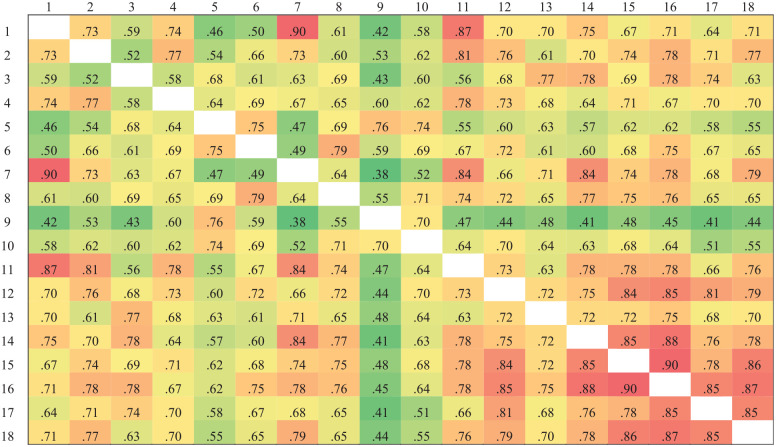
Cosine Similarity Results of the Anxiety Subscale

**Figure 3:**
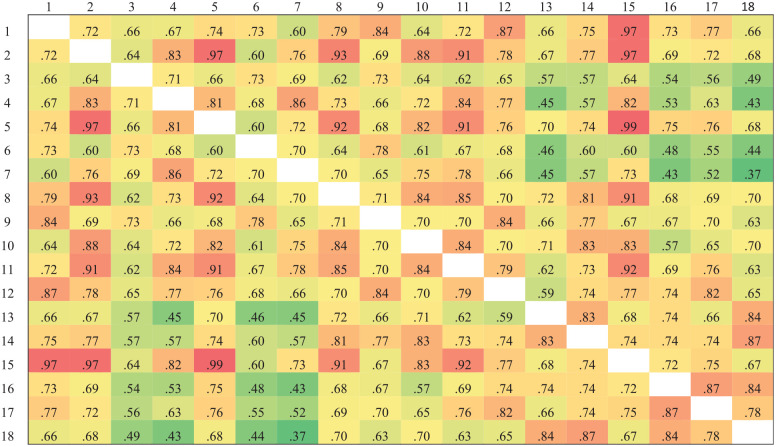
Cosine Similarity Results of the Avoidance Subscale

Figures 2 and 3 present the cosine similarity results for the two subscales. We calculated the mean cosine similarity of each item with all other items within the same subscale to obtain the item semantic similarity indices. Lower semantic similarity indices indicate that an item is more semantically unique. Based on these indices, we ranked the 18 items in each subscale from lowest to highest. The nine items with the lowest semantic similarity indices—representing the most semantically unique items—were selected for inclusion in the short form of the scale.

### Comparing the Scale Abbreviation Approaches

To address our research questions, we conducted Item Response Theory (IRT) analyses using the ECR subscales. Specifically, we employed the Graded Response Model (GRM; Samejima, 1969) to estimate item parameters (discrimination and thresholds), ability parameters, and the Test Information Function (TIF) utilizing the mirt package (Chalmers, 2012) in *R* (R Core Team, 2023). Each subscale was analyzed separately. As a result, we obtained one discrimination parameter and four threshold parameters for each item in both the avoidance and anxiety subscales. We used Spearman correlation coefficients to examine the relationships between the item parameters (discrimination and thresholds) and the item semantic similarity indices.

Next, we evaluated the TIF values to determine the levels of ability at which the test information was maximized for the subscales abbreviated using the ant colony optimization, genetic algorithms, and sentence embedding approaches. Furthermore, we computed Pearson correlations between the ability estimates obtained from the original scale and those from the different short forms created using the various scale abbreviation methods. This allowed us to assess the consistency of ability estimation across the original and abbreviated scales.

Finally, we conducted CFA to evaluate the model fit of both the original and abbreviated forms of the ECR scale. We utilized the lavaan package (Rosseel, 2012) in *R* (R Core Team, 2023), employing the DWLS estimation method. For assessing model fit, we examined several widely accepted indices: CFI, TLI, and the Standardized Root Mean Square Residual (SRMR). According to the criteria established by Hu and Bentler (1999), a good model fit is indicated by CFI and TLI values equal to or greater than 0.95, and an SRMR value less than or equal to 0.08. These thresholds are commonly used in psychometric evaluations to determine the adequacy of the factor structure.

We compared the model fit indices of the original ECR scale with those of each abbreviated form derived from the ant colony optimization, genetic algorithms, and sentence embedding approaches. This comparison allowed us to assess whether the abbreviated scales maintained the structural integrity and factorial validity of the original instrument. Ensuring that the abbreviated scales replicate the original factor structure is crucial for their validity and reliability in measuring attachment-related anxiety and avoidance. By employing CFA and these fit indices, we aimed to verify that the abbreviated versions not only reduced the number of items but also preserved the essential psychometric properties of the ECR scale. This analysis was instrumental in determining the effectiveness of our sentence embedding approach compared to traditional scale abbreviation methods.

## Results

### Relationships Between Item Parameters and Semantic Similarity

Discrimination parameters estimated from the GRM ranged from 1.55 to 2.58 for the avoidance subscale and from 1.15 to 2.24 for the anxiety subscale. According to Baker’s (2001) guidelines, discrimination parameters can be interpreted as follows: very low: 0.01 to 0.24, low: 0.25 to 0.64, moderate: 0.65 to 1.34, high: 1.35 to 1.69, and very high: 1.70 and above. Based on these criteria, items in the avoidance subscale exhibited high to very high discrimination indices, while items in the anxiety subscale ranged from moderate to very high discrimination.

Table 1 presents the relationships between discrimination parameters, item thresholds, and item semantic similarity indices. The correlations between item thresholds and the mean item semantic similarity indices were not significant for either subscale. This suggests that the position of the item on the latent trait continuum (as indicated by thresholds) is not related to its semantic similarity with other items. However, noteworthy findings emerged when examining the relationship between discrimination parameters and item semantic similarity indices. For the avoidance subscale, there was no significant correlation between the discrimination parameters and the mean item semantic similarity indices. In contrast, for the anxiety subscale, there was a significant negative correlation between the discrimination parameters and mean item semantic similarity indices (*r* = –.546, *p* < .05).

**Table 1:** The Relationships Between Discrimination Parameters, Item Thresholds, and Item Semantic Similarity Indices

Parameters	Semantic Similarity Index for Anxiety	Semantic Similarity Index for Avoidance
a	−.546*	.036
b1	−.298	.052
b2	−.107	−.080
b3	.109	−.011
b4	.295	.028

* *p* < .05.

This significant negative correlation in the anxiety subscale indicates that items with lower semantic similarity indices—that is, items that are more semantically unique compared to other items—tend to have higher discrimination parameters. In other words, semantically unique items are more effective at distinguishing between individuals with different levels of attachment-related anxiety. This finding underscores the potential utility of item semantic similarity indices in predicting item discrimination parameters, particularly for the anxiety subscale. Incorporating semantic similarity measures into the item selection process could enhance the scale abbreviation by identifying items that are both semantically unique and highly discriminative. This approach aligns with the goal of creating an abbreviated form that maintains the psychometric integrity of the original scale while reducing redundancy and participant burden.

### TIF for the Abbreviated Scales

**Figure 4:**
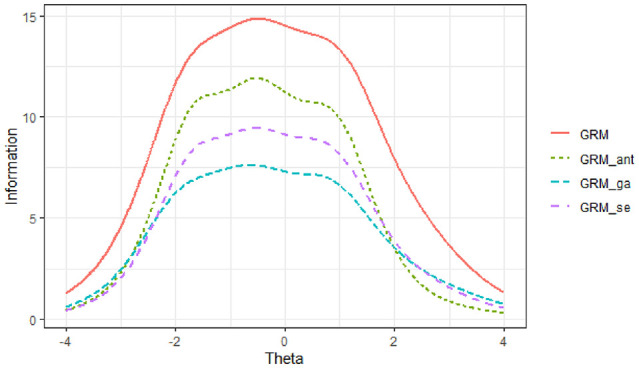
Test Information Function for the Anxiety Subscale *Note.* GRM: Graded Response Model results from the original scale; GRM_ant: Graded Response Model results obtained from ant colony optimization scale abbreviation approach; GRM_ga: Graded Response Model results obtained from genetic algorithms scale abbreviation approach; GRM_se: Graded Response Model results obtained from sentence embedding scale abbreviation approach.

**Figure 5:**
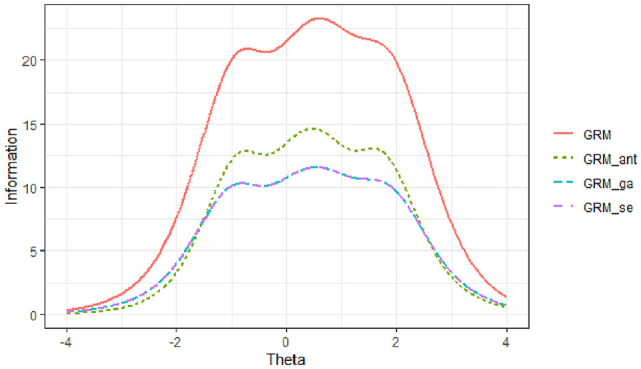
Test Information Function for the Avoidance Subscale *Note.* GRM: Graded Response Model results from the original scale; GRM_ant: Graded Response Model results obtained from ant colony optimization scale abbreviation approach; GRM_ga: Graded Response Model results obtained from genetic algorithms scale abbreviation approach; GRM_se: Graded Response Model results obtained from sentence embedding scale abbreviation approach.

Across all scale abbreviation methods, the TIFs for the anxiety subscale were maximized between ability levels of θ = −2 and θ = 1, while for the avoidance subscale, the TIFs peaked between the ability levels of θ = −1 and θ = 2 (see Figures 4 and 5). The results indicated that the TIFs obtained from the three scale abbreviation approaches were very similar across the ability range for each subscale. Among these methods, the TIF derived from the ant colony optimization approach most closely resembled the original TIF for both subscales. Furthermore, the findings showed that compared to the genetic algorithms approach, the TIF from the sentence embedding approach more closely matched the original TIF for the anxiety subscale. For the avoidance subscale, the TIFs from the sentence embedding and genetic algorithms approaches were identical, both aligning closely with the original TIF.

These results suggest that all three abbreviation methods effectively preserved the informational properties of the original scales within their respective ability ranges. However, the ant colony optimization approach demonstrated the highest fidelity to the original TIFs across both subscales. The sentence embedding approach showed comparable performance, especially for the anxiety subscale, indicating its potential effectiveness in maintaining the psychometric integrity of the scale while also considering semantic uniqueness.

### Model Fit Comparison

Table 2 presents the model fit results for the three scale abbreviation approaches: genetic algorithms, ant colony optimization, and sentence embedding. The ant colony optimization method demonstrated an excellent fit to the data, with SRMR = 0.054, CFI = 0.986, and TLI = 0.984, indicating a very close alignment between the model and the observed data. Similarly, the genetic algorithms approach showed good model-data fit, with SRMR = 0.093, CFI = 0.951, and TLI = 0.944. The sentence embedding approach also yielded acceptable fit indices, with SRMR = 0.080, CFI = 0.961, and TLI = 0.956. Although the ant colony optimization approach achieved the best overall fit, the differences in model fit indices among the three methods were relatively small. All three approaches produced fit indices that meet or exceed the commonly accepted thresholds for good model fit (Hu & Bentler, 1999), namely CFI and TLI values of 0.95 or higher and SRMR values of 0.08 or lower. This suggests that each abbreviation method effectively maintained the factorial structure and psychometric integrity of the original ECR scale. Furthermore, the similarity in model fit indices across the three methods indicates that the sentence embedding approach is a viable alternative for scale abbreviation, offering comparable model fit while also considering semantic uniqueness of items.

**Table 2:** Model Fit Results Based on the Genetic Algorithm, Ant Colony Optimization, and Sentence Embedding Approaches.

Scale abbreviation approach	CFI	TLI	SRMR
Original scale	.948	.945	.097
Ant colony optimization	.986	.984	.054
Genetic algorithm	.951	.944	.093
Sentence embedding	.961	.956	.087

### Relationships Between Ability Estimates

Table 3 presents the correlations among the estimated ability parameters obtained from the three short forms derived using the genetic algorithms, ant colony optimization, and sentence embedding approaches. All estimated ability levels from these abbreviation methods showed high correlations with those from the original scale, indicating that each method effectively reproduces the original ability estimates. Specifically, the ability estimates from the genetic algorithm approach exhibited the highest correlations with those from the original scale. Overall, these findings suggest that utilizing the sentence embedding method or automatic item selection procedures (i.e., genetic algorithms and ant colony optimization) does not significantly affect the students’ relative positions within the sample. This implies that the abbreviated scales, regardless of the method used, maintain the measurement properties of the original scale and effectively preserve the rank order of individuals.

**Table 3:** Correlations Between Estimated Ability Parameters Based on the Genetic Algorithm, Ant Colony Optimization, and Sentence Embedding Approaches

	Anxiety			Avoidance		
Scaleabbreviationapproach	Originalscale	Geneticalgorithm	Antcolonyoptimization	Originalscale	Genetic algorithm	Antcolonyoptimization
Geneticalgorithm	.976***			.982***		
Ant colony optimization	.949***	.946***		.970***	.962***	
Sentenceembedding	.964***	.952***	.962***	.982***	1***	.962***

*** *p* < .001.

**Figure 6:**
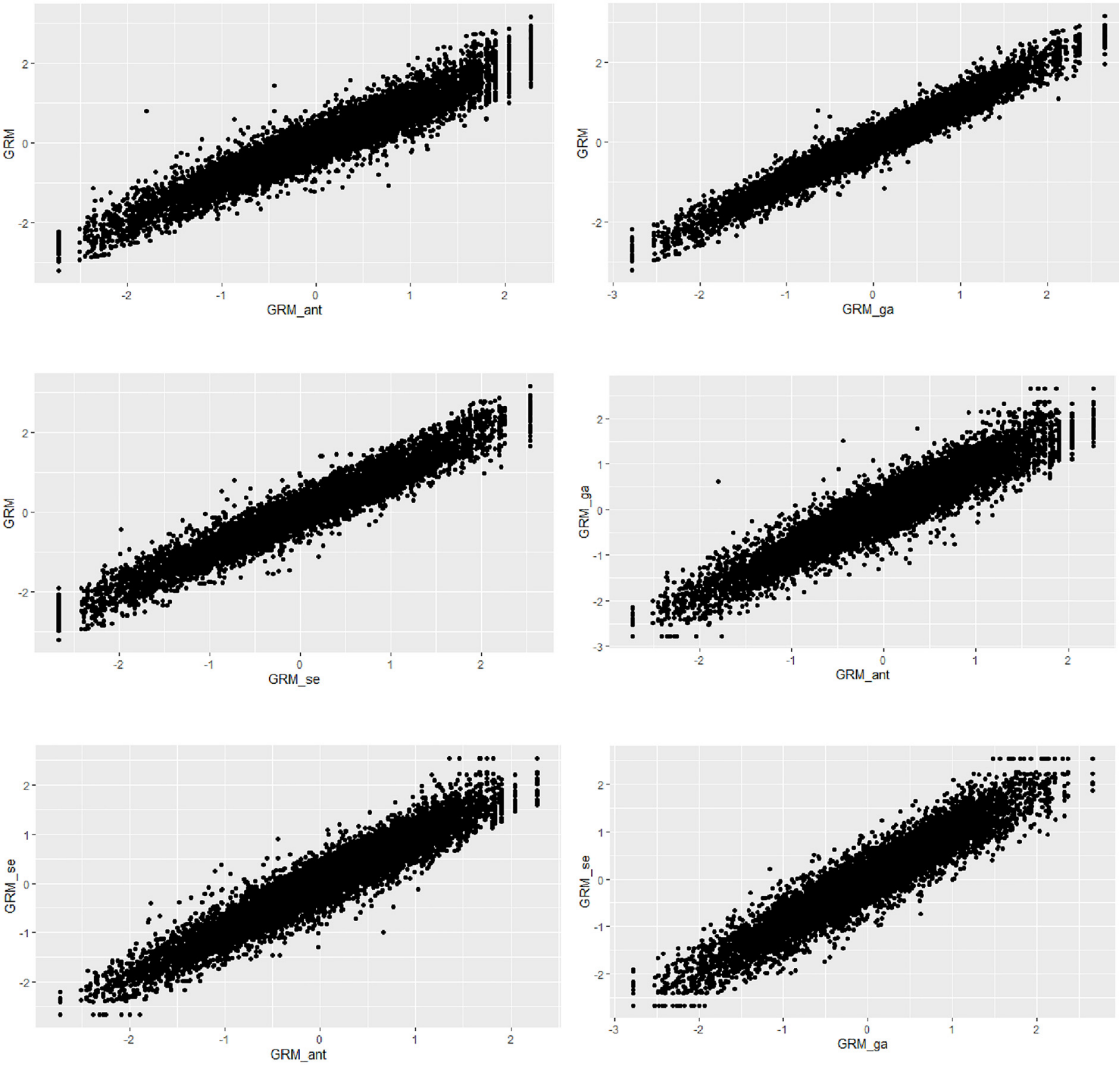
The Relationship Between the Estimated Ability Values for the Anxiety Subscale by the Scale Abbreviation Approaches *Note.* GRM: Graded Response Model results from the original scale; GRM_ant: Graded Response Model results obtained from ant colony optimization scale abbreviation approach; GRM_ga: Graded Response Model results obtained from genetic algorithms scale abbreviation approach; GRM_se: Graded Response Model results obtained from sentence embedding scale abbreviation approach.

**Figure 7:**
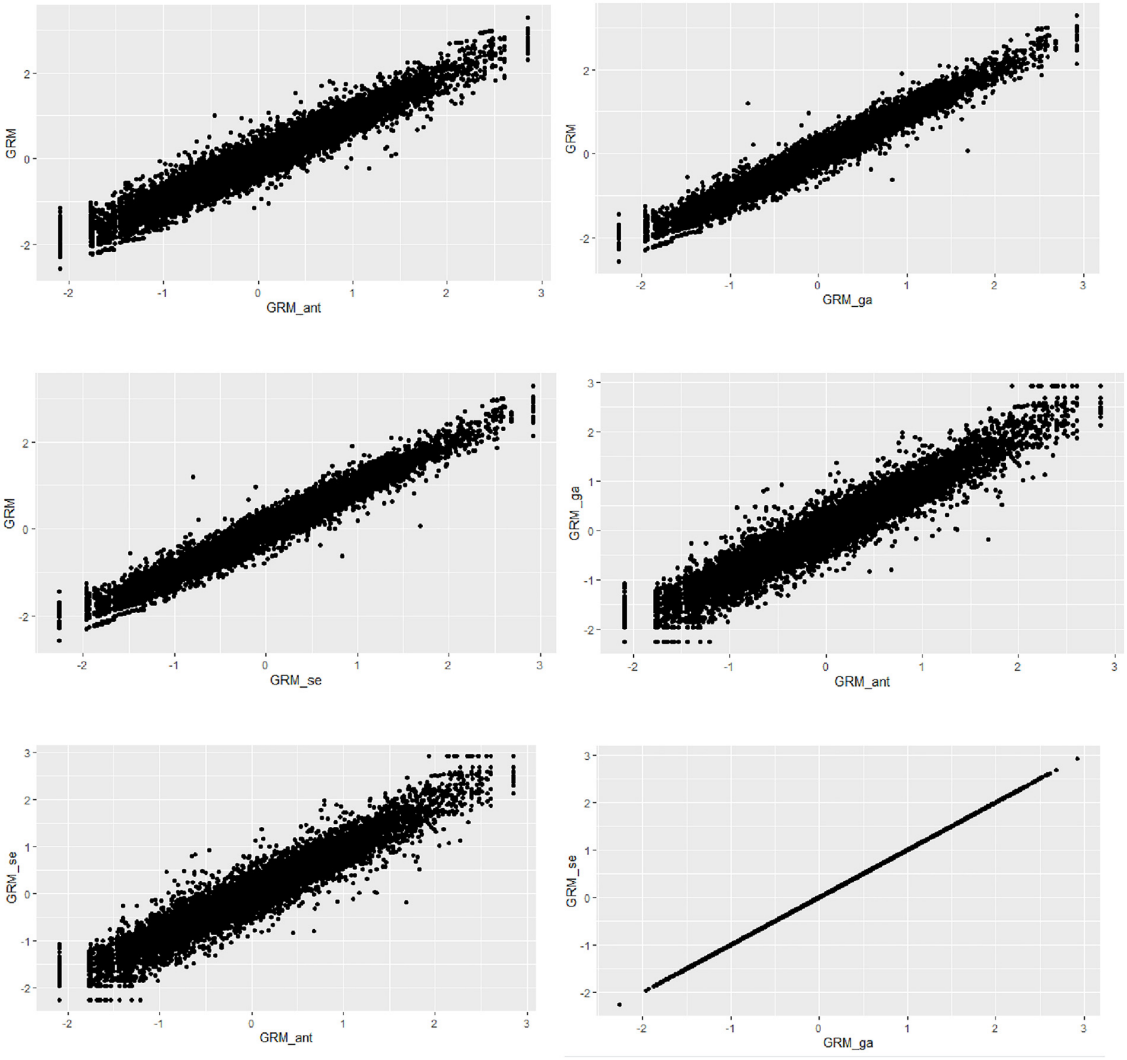
The Relationship Between the Estimated Ability Values for the Avoidance Subscale by the Scale Abbreviation Approaches *Note.* GRM: Graded Response Model results from the original scale; GRM_ant: Graded Response Model results obtained from ant colony optimization scale abbreviation approach; GRM_ga: Graded Response Model results obtained from genetic algorithms scale abbreviation approach; GRM_se: Graded Response Model results obtained from sentence embedding scale abbreviation approach.

The relationships between the ability estimates obtained from all three scale abbreviation approaches are shown in Figures 6 and 7. The findings suggest strong linear relationships between the ability estimates derived from the three different scale abbreviation methods. However, an intriguing result emerged when examining the correlation between the ability estimates from the genetic algorithms and sentence embedding approaches for the avoidance subscale. Despite employing different item selection algorithms, the correlation between the ability estimates from these two methods was perfect (i.e., *r* = 1). This indicates that both methods selected the same items although genetic algorithms as a data-driven method relied entirely on the response dataset and the sentence embedding approach focused solely on semantic similarity among the items without considering item responses.

## Discussion

In recent years, automatic scale abbreviation methods have gained popularity for creating short forms of psychological assessment scales due to their numerous advantages (Smith et al., 2000; Stanton et al., 2002). However, these data-driven methods typically require administering the full scale to a relatively large sample that represents the target population to be effective (Olaru et al., 2015). In certain contexts, such as clinical settings, obtaining sufficient sample sizes for both pretesting and final administration may not be feasible (Furr, 2011). To address this limitation, our study proposed a scale abbreviation approach based on sentence embedding, which we compared to two established automatic scale abbreviation methods. This new approach allows researchers to abbreviate lengthy scales more effortlessly by leveraging items’ semantic similarities without the need for pretest data. In addition, this method could be valuable in scale development studies, offering a practical alternative to traditional data-based approaches (Pennington et al., 2014).

Psychological testing aims to accurately determine individuals’ levels on various psychological trait continuums (Embretson & Reise, 2000). Therefore, it is crucial to abbreviate scales without significant loss of information. In this study, we proposed a sentence embedding approach that performed comparably to automatic scale abbreviation methods, providing high measurement accuracy across a wide range of ability levels. CFA results also supported the similarity between the sentence embedding approach and other abbreviation methods in terms of model fit. Furthermore, we found that ability estimates derived from the sentence embedding approach closely mirrored those obtained from the original scale, aligning with findings from previous studies utilizing semantic methods (Hsu et al., 2018).

Unlike other scale abbreviation methods, the sentence embedding approach does not require pre-administration of the scale to select items for the short form. Instead, it uses items’ semantic similarities for item selection. Since data-driven methods, such as ant colony optimization and genetic algorithms, might inadvertently select semantically similar items for the abbreviated form, intentionally choosing semantically dissimilar items is a reasonable strategy to maximize content coverage and maintain variance (Larsen & Bong, 2016). Participants tend to respond similarly to semantically similar items, so selecting distinct items can enhance the scale’s discriminative power without sacrificing reliability (Clark & Watson, 1995).

An interesting finding of our study was the significant moderate negative correlation between item discrimination parameters and the mean semantic similarity indices for the anxiety subscale. This suggests that semantically unique items tend to have higher discrimination parameters, supporting previous research indicating associations between the semantic and psychometric features of items (Hsu et al., 2018; Mitkov et al., 2009). These results imply that semantic features might eventually replace the need for extensive pretesting in scale development. However, this relationship was not observed for the avoidance subscale, potentially due to the presence of reverse-scored items. In our analysis, a number of reverse-scored items were rephrased to align their semantic content, but this process may not fully capture the nuances of participants’ responses. For example, disagreeing with “I feel comfortable sharing my private thoughts and feelings with my partner” does not necessarily equate to agreeing with “I do not feel comfortable sharing my private thoughts and feelings with my partner.” Future studies could explore the impact of reverse-scored items by comparing both negative and positive statements in terms of semantic and psychometric properties (Weems et al., 2003).

In conclusion, our study demonstrates that the sentence embedding approach to scale abbreviation yields results comparable to other automatic methods in terms of model fit, TIF, and the relationship between ability estimates from the original and abbreviated scales. Also, the moderate negative correlation between semantic similarity and discrimination parameters in the anxiety subscale highlights the predictive value of semantic uniqueness in item selection. These findings underline the potential of the sentence embedding approach to replace data-driven abbreviation methods that require a sufficient amount of response data collected from a representative group of respondents.

### Limitations and Future Research

As with many studies, our study also has certain limitations. Although we used the GRM within the IRT framework to compare scale results based on different abbreviation approaches, future studies could employ alternative IRT models, such as the Partial Credit Model (Masters, 1982), to have a more comprehensive comparison of the performance of the sentence embedding approach with other scale abbreviation methods. Furthermore, future studies could investigate the sentence embedding approach in scale abbreviation using both IRT and Classical Test Theory frameworks. Applying both frameworks may provide a more comprehensive evaluation of the method’s effectiveness and generalizability. Finally, while we observed a moderate negative correlation between the item semantic similarity averages and the discrimination parameters for the anxiety subscale, this relationship was not present for the avoidance subscale, which included many reverse-scored items (9 out of 18). Reverse-scored items might be a variable that affects this relationship. Therefore, future research is needed to examine whether the results of the sentence embedding approach predict the discrimination parameters when accounting for reverse-scored items. Furthermore, we aimed to conduct an in-depth analysis of BERT’s capabilities as a foundational step before extending our investigation to other methods. Future research could extend this work by incorporating a comparative analysis of BERT with other transformer-based methods to further validate and expand the findings.

## Appendix A

**Table:** List of Items of ECR

1. I prefer not to show a partner how I feel deep down.
2. I worry about being abandoned.
3. I am very comfortable being close to romantic partners.
4. I worry a lot about my relationships.
5. Just when my partner starts to get close to me, I find myself pulling away.
6. I worry that romantic partners won’t care about me as much as I care about them.
7. I get uncomfortable when a romantic partner wants to be very close.
8. I worry a fair amount about losing my partner.
9. I don’t feel comfortable opening up to romantic partners.
10. I often wish that my partner’s feelings for me were as strong as my feelings for him/her.
11. I want to get close to my partner, but I keep pulling back.
12. I often want to merge completely with romantic partners, and this sometimes scares them away.
13. I am nervous when partners get too close to me.
14. I worry about being alone.
15. I feel comfortable sharing my private thoughts and feelings with my partner.
16. My desire to be very close sometimes scares people away.
17. I try to avoid getting too close to my partner.
18. I need a lot of reassurance that I am loved by my partner.
19. I find it relatively easy to get close to my partner.
20. Sometimes I feel that I force my partners to show more feeling, more commitment.
21. I find it difficult to allow myself to depend on romantic partners.
22. I do not often worry about being abandoned.
23. I prefer not to be too close to romantic partners.
24. If I can’t get my partner to show interest in me, I get upset or angry.
25. I tell my partner just about everything.
26. I find that my partner(s) don’t want to get as close as I would like.
27. I usually discuss my problems and concerns with my partner.
28. When I’m not involved in a relationship, I feel somewhat anxious and insecure.
29. I feel comfortable depending on romantic partners.
30. I get frustrated when my partner is not around as much as I would like.
31. I don’t mind asking romantic partners for comfort, advice, or help.
32. I get frustrated if romantic partners are not available when I need them.
33. It helps to turn to my romantic partner in times of need.
34. When romantic partners disapprove of me, I feel really bad about myself.
35. I turn to my partner for many things, including comfort and reassurance.
36. I resent it when my partner spends time away from me.

## Notes

1.
https://openpsychometrics.org
